# Effect of Telenursing on Supportive Care Needs in Patients with Melanoma and Lung Cancer on Targeted Therapies: A Randomised Controlled Trial Study Protocol

**DOI:** 10.3390/mps7050078

**Published:** 2024-10-03

**Authors:** Aurora De Leo, Gloria Liquori, Alessandro Spano, Nicolò Panattoni, Sara Dionisi, Laura Iacorossi, Noemi Giannetta, Irene Terrenato, Emanuele Di Simone, Marco Di Muzio, Fabrizio Petrone

**Affiliations:** 1 Department of Biomedicine and Prevention, University of Rome Tor Vergata, 00133 Rome, Italy; aurora.deleo@ifo.it (A.D.L.); gloria.liquori@uniroma1.it (G.L.); alessandro.spano@ifo.it (A.S.); 2 Nursing Research Unit IFO, IRCCS Istituti Fisioterapici Ospitalieri, 00144 Rome, Italy; 3 Department of Public Health and Infectious Diseases, Sapienza University of Rome, 00185 Rome, Italy; emanuele.disimone@uniroma1.it; 4 Nursing, Technical, Rehabilitation Department, DaTeR Azienda Unità Sanitaria Locale di Bologna, 40124 Bologna, Italy; sara.dionisi@uniroma1.it; 5 Department of Life, Health and Health Professions Sciences, Link Campus University, 00165 Rome, Italy; l.iacorossi@unilink.it; 6 Departmental Faculthy of Medicine, Saint Camillus International University of Health and Medical Sciences (UniCamillus), 00131 Rome, Italy; noemi.giannetta@unicamillus.org; 7 CTC and Biostatistics and Bioinformatics—Scientific Direction, IRCCS Regina Elena National Cancer Institute, 00144 Rome, Italy; irene.terrenato@ifo.it; 8 Department of Clinical and Molecular Medicine, Sapienza University of Rome, 00185 Rome, Italy; marco.dimuzio@uniroma1.it; 9 Nursing, Technical, Rehabilitation, Assistance and Research Direction, IRCCS Istituti Fisioterapici Ospitalieri, IFO, 00144 Rome, Italy; fabrizio.petrone@ifo.it

**Keywords:** lung, melanoma, neoplasms, randomised controlled trial, nurse, study protocol, supportive care needs, targeted therapy, telenursing

## Abstract

**Background**: Telenursing comprises a set of tools and interventions enabling nurses to provide remote care. This study aims to assess the impact of telenursing interventions on the supportive care needs of patients with melanoma and lung cancer who are receiving targeted therapies. **Methods**: This six-month monocentric, double-arm, randomised, controlled trial study protocol will assess the effect of telenursing on the supportive care needs (primary outcome) in 40 patients (20 in each group) after one month. The secondary outcomes will be monitored at baseline, one, three and six months: supportive care needs (at three and six months), therapeutic adherence, quality of life, usability and satisfaction, performance status, patient-reported outcomes and main adverse events. The SPIRIT guidelines will be used for the reporting. **Results**: The results from this trial will assess the impact of a telenursing intervention on cancer care. **Conclusions**: This trial could be a starting point for more extensive studies on telenursing interventions to promote nurses’ skills, as well as the quality and safety of care in patients with cancer, highlighting the impact of more outstanding nursing contributions on cancer care. **Trial and Protocol Registration**: The study protocol was approved by the relevant Italian Ethics Committee Lazio Area 5 (RS1851/23, 2773; 6 September 2023) and was registered on ClinicalTrials.gov (trial registry number NCT06254196).

## 1. Introduction

According to the GLOBOCAN 2018 classification [1], lung cancer is the leading cause of cancer mortality, and melanoma is the third most commonly diagnosed cancer in the United States of America (USA) [2]. The study of the molecular characteristics of diseases has allowed targeted therapies (TT) to have more excellent selectivity in terms of the therapeutic target by reducing the traditional toxicities of chemotherapy [3]. The daily home intake of these chemotherapy drugs for a long time is related to the onset of specific adverse events (AEs) and needs. Especially in the first months of treatment, AEs significantly impact patients’ health-related quality of life (HRQoL) [4], sometimes more critically than traditional chemotherapy [5]. Supportive care, which is focused on supporting the complex physical, psychological and social needs related to cancer and its treatments, is considered a practical approach to the adaptation process of patients with cancer [6]. The international literature emphasises the crucial role of the multidisciplinary team, the proactive support of nurses, and the use of patient-reported outcomes (PROs) in improving treatment adherence, early prevention, diagnosis, and correct management of treatment-related AEs, QoL and optimising healthcare resources in cancer care [7,8,9]. Therefore, face-to-face and digital–remote interventions by nurses (telenursing) are recommended to promote cancer patients’ self-care, self-efficacy, clinical outcomes and continuity of care [8,9]. Specifically, telenursing refers to the remote tools and interventions used by nurses to improve the quality of and access to care, communication and exchange of information between healthcare professionals and patients [8]. Lately, in cancer care, several studies have also been conducted using telenursing interventions to manage treatment-related demands and toxicities. In particular, recent studies show that telenursing has a positive impact on the prevention and management of fatigue, anxiety, depression and symptom distress [10,11]. However, despite an increase in telenursing interventions in recent years [12], further studies are needed to test the impact of telenursing interventions on managing patients’ needs, AEs, QoL and PROs [13,14]. A real-time monitoring system could im-prove transitional care, positively impacting the quality of care, patient safety and healthcare resource utilisation [11,15].

To promote the generalisability of the results, considering their incidence and common therapeutic approaches, patients with melanoma and lung cancer were included in this study [16,17].

Furthermore, due to the prevalence and the high burden of lung cancer and melanoma in patients on TT, comparing a telenursing intervention with standard care could highlight its efficacy in cancer care.

### 1.1. Aim

This randomised controlled trial (RCT) study protocol aims to reduce the treatment-related burden of patients with lung cancer and melanoma on TT through increased nursing involvement and the use of telenursing, improving their clinical pathway.

### 1.2. Primary and Secondary Objectives

The objectives of this study will be measured in two compared groups.

Primary objective: To assess the impact of telenursing interventions on the supportive care needs (SCNs) [18,19] in patients with lung cancer and melanoma at the first prescription of TT after one month of treatment (T1). Secondary Objectives: To explore the differences between the AEs reported by healthcare professionals and the patient-reported outcomes and develop a specific nursing reporting system to improve the quality, safety and continuity of cancer care. Further secondary outcomes are shown in Table 1, together with the tools, timing, and users involved in the data collection. Among the secondary outcomes, the SCNs over time (until the end of the study) [18,19], quality of life [20,21], patient satisfaction [22,23], therapeutic adherence [24,25] and performance status [26] will be assessed and compared in both groups. Moreover, the incidence and severity of the following vital signs and adverse events will be compared to highlight the effectiveness of the telenursing intervention with tools based on the Common Terminology Criteria for Adverse Events (CTCAE) [27]: body temperature, blood pressure, heart rate, body weight, pain, stomatitis and mucositis, dysgeusia, nausea and vomiting, diarrhoea, loss of appetite, fatigue, headache, dermatologic reactions, cough and dyspnoea, pain, palmar–plantar erythrodysesthesia, bleeding events, photosensitivity, ophthalmological reactions, sleep, mood and voice disorders.

## 2. Materials and Methods

The Standard Protocol Items Recommendations for Interventional Trials (SPIRIT) checklist [28] was used to report this study protocol (Supplementary Table S1).

Supplementary Table S1. Standard Protocol Items Recommendations for Interventional Trials (SPIRIT) checklist.

### 2.1. Experimental Design

A six-month, double-arm, prospective, monocentric, RCT will be performed to assess the impact of a telenursing intervention on the reduction of the treatment-related burden in patients with lung cancer and melanoma on TT, improving their clinical pathway. The telenursing intervention will be added to clinical practice (intervention arm, IA) to highlight differences in the study’s outcomes compared to the control group (control arm, CA), which was followed according to clinical practice.

### 2.2. Study Setting and Sampling

The RCT will be performed in a national cancer centre starting on 1 April 2024. A nursing team will lead the project in collaboration with medical oncologists. The sample size calculation was performed on the primary outcome, the SCNs scale [18,19] measured one month after treatment, considering possible losses to follow-up (drop-out rate). The study by Ebrahimabadi [18] allowed the researchers to hypothesise the following:

- A mean score and its relative standard deviation (SD) in the intervention group equal to 81.53 (±13.2),
- A mean score and its relative SD in the control group equal to 103.5 (±21.96), with a statistical power of 90% and a significance level α = 0.05, while a two-tailed Student’s *t*-test estimated an overall size of 32 patients, 16 for each group. Given the mean score reported in the hypothesis system, we obtain an effect size equal to 1.21. Considering a drop-out rate of 20%, we will enrol a total sample of 40 patients. The software NCSS PASS v.11.0 was used to assess the minimum size. Evaluation of the statistical tests will be carried out by an expert statistician.

### 2.3. Inclusion and Exclusion Criteria

Inclusion criteria: oncological outpatients with melanoma and lung cancer, 18–75 years old, on the first prescription of TT; able to understand and speak the national language; patients with internet access and a personal computer, or smartphone or tablet device; and able to sign an informed consent and willing to adhere to the procedures of the study. Exclusion criteria: patients affected by other malignancies, previously treated with TT; patients with concomitant cancer treatment (chemotherapy, radiation therapy, immunotherapy, and exclusive palliative care) and patients with poor compliance interfering with study procedures. The exclusive inclusion of patients naïve to TT aims to reduce the potential bias related to the effect of previous educational interventions to prevent and monitor care-related needs. Nurses and oncologists will introduce the study to all eligible patients of the oncological department during the follow-up visits. Eligible patients will be given a copy of the informed consent, setting a subsequent appointment within 7 days for the enrolment visit (baseline) and agreeing to formal adherence to the study by signing the aforementioned informed consent.

### 2.4. Detailed Procedure

Two research nurses (PhD and PhD student) with previous experience in telenursing and cancer treatment with TT [11,29] will be involved from the patient enrolments to the end of the study. The research nurses involved will introduce the project to interested patients, explaining the objectives, purposes, and procedures of the study and the implications for clinical practice.

They will provide the following:

- Nursing assessment: to allow comparison between the two groups, the questionnaires described in Table 1 will be used at baseline and according to the timing described in Table 1. The measurements will allow researchers to highlight the effectiveness of the telenursing intervention and the improvement of patients’ clinical path.
- Support in study procedures: during the 6-month follow-up, nurses will provide advice to the patients involved to improve the experience with the intervention and the tools used, and also in order to optimize the data collection in the two compared groups.
- Intervention in the IA: with patients involved in the experimental group, research nurses will use telenursing to monitor the supportive care needs and other objectives described in Section 1.2. For the purposes of the study, the telenursing intervention will use the Intelligence2health platform for video calls, a dedicated telephone line, short messages and electronic tools. During the 6-month study, nurses will implement a total of 8 video calls. Nurses will implement patient education interventions to improve patient care, quality of life, satisfaction, treatment adherence, problem-solving skills, and self-care. Suggestions to better manage patients’ needs will be evidence-based and may be agreed with medical oncologists as needed. However, they will be kept constantly updated on the health status of patients. Nursing interventions will be aimed at promoting the care needs of patients.
- Data collection will be carried out using the chosen tools, according to the timing described in Table 1, at baseline, daily, after 30, 90 and 180 days, to allow comparison between the intervention arm and control arm. In particular, patients and nurses will monitor the burden and needs related to care through the Supportive Care Needs scale [18,19]; therapeutic adherence through the MARS-5I [24,25]; QoL through the QoL SF-36 [20,21]; satisfaction and usability of the system through the SUS [22,23]; performance status through the Karnofsky performance status [26]; and the AEs described in Section 1.2 through the CTCAE tools [27]. Patients enrolled in the control group will use paper questionnaires, while patients in the intervention group will use electronic tools that nurses will check daily to implement any coaching interventions. Any changes in the health status or needs of patients can be intercepted early thanks to the filling out of daily forms by the patients themselves, which nurses will check daily.
- Creation and management of a specific database.

After nurses have obtained the informed consent, 40 patients with melanoma and lung cancer (20 in each group) will be randomised into the IA (telenursing intervention added to clinical practice) or the CA (clinical practice). To allow for comparison, at baseline (T0) before the randomisation, patients from both groups will complete the tools shown in Table 1 in paper format. Additionally, patients involved in the study will use the study tools according to the timelines described in Table 1 at T1 (30 days), T2 (90 days), and T3 (180 days). Regarding the practice procedures, monthly telenursing interventions will be provided every 30 days, on dates agreed in advance with patients. However, patients will be able to communicate with the nursing staff via short messages or by telephone at any time of the day, between 8 a.m. and 2 p.m. An unplanned nursing intervention can be useful in case of patient doubts or problems related to therapeutic adherence (dosage errors, forgotten or double doses, etc.), onset of new side effects, sudden alterations in vital signs, etc. In addition, in view of the daily questionnaire filled out by patients (Table 1), nursing staff will implement additional interventions as needed, remotely or in person.

#### 2.4.1. Control Arm

The CA patients will attend monthly visits with oncologists and receive in-person nursing care as needed. They will fill out paper-form questionnaires collected by nurses during the monthly visits until the end of the study. Nurses and oncologists will not consider patient data during follow-up, and according to clinical practice, patients should contact oncologists as needed. Data collected by nurses during the monthly visits and by patients at home will be recorded in a specific database by the nurses, but these data will not be evaluated by nurses or oncologists during the study. However, according to current clinical practice, if necessary, patients in the control group will be able to contact oncologists and nurses via the telephone and email addresses provided before the start of the study.

#### 2.4.2. Intervention Arm

In addition to clinical practice, after the randomisation, the IA patients will receive an educational nurse intervention to improve the SCNs [18,19], prevent the main AEs and recap the study procedures. Furthermore, at baseline, the IA patients will receive the following:

- A link to access the electronic questionnaires through a web platform (GDPR and privacy compliant) (daily, at one, three and six months).
- A link to access the platform Intelligence2health [30], actually used in the same national cancer centre for the monthly telenursing interventions.
- A company telephone number to contact the team nurses.

In the first month, nurses involved in the study will carry out two telenursing interventions through a video call enabled by the platform (Table 2). The first virtual meeting will be conducted seven days after baseline (T0), focusing on the study procedures and e-tools. Subsequent telenursing interventions will be carried out monthly. Research nurses will review the daily data from the IA patients to arrange appropriate reinforcement interventions (coaching), agreed upon with oncologists where applicable. However, the oncologists will be steadily updated on the patients’ clinical conditions. The telenursing interventions will aim to prevent, monitor and improve treatment-related outcomes (Table 1). Specifically, evidence-based nursing coaching interventions will aim to improve the SCNs [18,19], adverse events and vital signs described in the primary and secondary objectives, and what will be required by patients regarding treatment-related needs.

### 2.5. Fidelity of Intervention and Randomisation

Nurses will prevent and manage both groups of patients’ toxicities and needs according to good clinical practice [31], in collaboration with the oncologists.

The random sequence of eligible patients in the CA and the IA will be 1:1. The assignment list will be generated by special software before the study starts, and it will be unknown to the nurses and the oncologists. Due to the nature of the interventions, the patients and healthcare professionals will not be blinded. In case of system dysfunctions or desire to leave the study, all the patients will be followed according to the current clinical practice by healthcare teams.

Patients may drop out of the study at any time without affecting their care pathways, which will continue to be provided according to current clinical practice. At the same time, patients who do not adhere to the study procedures may be excluded from the study after 15 days of missing data.

### 2.6. Data Collection

The planning of the study will include an enrolment period lasting 12 months, starting from the involvement of the first patient after 15 June 2024. The study follow-up will last six months. After this period, all the patients will be referred to current clinical practice. Nurses will record digital data from patients’ self-reports and nurses’ interviews and observations in a specific database.

Patients in the control arm will be treated according to current clinical practice, mainly with the support of oncologists. The paper questionnaires that patients and nurses will complete during the study will be recorded by nurses in a specific database.

Data from patients enrolled in the intervention group will be collected via electronic questionnaires, similar to those of nurses. Data collection for the outcomes described in Table 1 will be assessed at baseline (T0), after 30 (T1), 90 (T2) and 180 days (T3). In addition, patients will transmit data daily relating to the monitoring of vital parameters and AEs described in the section on the primary and secondary objectives.

### 2.7. Data Analysis

Intention-to-treat analysis will be applied in case of abandonment of a patient from one arm or the other. The rigor observed in the study will be aimed at acquiring detailed and exhaustive information. However, missing data due to incomplete or partial registration by the patient will be treated with appropriate statistical techniques. Descriptive statistics will explore the continuous variables through the means and standard deviations (±SD), while the categorical variables will explore the absolute frequencies and percentage values. The Kolmogorov–Smirnov normality test will be used for all the continuous variables. The Chi-square or Fisher’s exact test will assess the potential associations between categorical variables. The differences for continuous variables will be explored using the nonparametric Mann–Whitney U test or Student’s *t* test, depending on the nature of the distributions of the variables examined. All the statistical analyses will be performed using Statistical Package for the Social Sciences (SPSS) Software 29.0 (IBM SPSS Statistics for Windows, Version 29.0. Armonk, NY, USA: IBM Corp.) for Windows. A *p*-value < 0.05 will be considered statistically significant.

### 2.8. Ethical Considerations

The study protocol was approved by the relevant Ethics Committee (RS1851/23, 2773, version v.0) and was recorded on ClinicalTrial.gov (identifier number NCT06254196, last update posted on 3 June 2024). Including patients in the project will be preceded by acquiring specific written informed consent. By signing the informed consent form, patients will formally agree to adhere to the procedures of the project study, thereby authorizing the authors to collect and analyse the data and disseminate the results to the scientific community. The data will be analysed in an aggregated and pseudo-anonymous form, and participants can only be identified by the research team. Moreover, data will be collected, stored, used, processed and transmitted in compliance with current legislation, guaranteeing its quality, integrity, security, availability and traceability following the original protocol. Possible changes to the protocol will be submitted to the committee for an appropriate opinion.

## 3. Expected Results

The results of this RCT could highlight the impact of an increased nursing contribution and telenursing interventions on SCNs [18,19] and cancer care.

The study protocol aims to assess the impact of a telenursing intervention in patients with lung cancer and melanoma at the first prescription of TT. In particular, this study aims to assess the impact of telenursing interventions on the SCNs [18,19] after one month of treatment (primary outcome) and improve the clinical pathways of patients with melanoma and lung cancer on TT. Regarding the safety of care, telenursing interventions aim to enhance clinical practice through a more outstanding nursing contribution with remote care. Furthermore, this study’s results could allow nurses to offer a more significant contribution within multidisciplinary teams in the therapeutic pathways of patients with cancer, as suggested by the previous literature, and improve their skills in this regard [29]. The importance of nurses’ contribution to remote care is inherent in its potential developments, present and future, for the discipline, the healthcare organizations and the community. In particular, as suggested by previous studies [32], this clinical trial could highlight, even in telenursing, the importance of the nurse–patient relationship, based on mutual trust, communication, common values and health objectives.

### 3.1. Strengths and Limitations of the Work

To the best of the authors’ knowledge, this is the first RCT to assess the impact of telenursing interventions on the clinical pathway of patients with melanoma and lung cancer on TT. In particular, it could be helpful to assess the effects of a broader nursing contribution on the SCNs [18,19] at one month (primary outcome), the main AEs, therapeutic adherence [24,25], QoL [20,21], system usability and satisfaction [22,23], performance status [26] and PROs at one, three and six months (secondary outcomes).

The authors are aware of the limitations of the study. First, the small sample size and the two cancer types enrolled (lung cancer and melanoma) should be considered. Furthermore, the lack of stratification by age and pathology may not allow the authors to capture differences or risk classes to consider through inference on a larger sample. The enrolment of patients in the exclusive TT does not allow for the inclusion of patients who probably could benefit from the interventions in the study. However, to reduce the weight of the limitations of the present study protocol, the authors trust in the methodological rigour of the study design.

### 3.2. Recommendations for Further Research and Practice

The results of the present study protocol should undoubtedly be tested on more extensive and heterogeneous samples and populations to assess the effectiveness and reliability in patients with cancer on TT.

### 3.3. Positionality Statements

Seven authors of this project are oncology nurses from a national cancer institute. The remaining four authors are experts in quantitative research methodology.

## 4. Conclusions

Telenursing interventions could significantly challenge nurses in improving cancer care outcomes [11]. Remote interactions promote patient–nurse coaching interventions to improve cancer patients’ self-care and problem-solving skills during chemotherapy. Nursing alerts for severe toxicities can enable early interventions to manage treatment-related issues, reducing interruptions and improving clinical outcomes [33]. Continuous monitoring could also enhance communication with the care team and highlight the reasons for non-adherence among cancer patients. The expected results could be used in implementing patient-centred telenursing interventions to be tested on larger populations affected by other treatments and illnesses.

## Figures and Tables

**Table 1:** Outcomes and tools.

Time	User	Outcomes	Tools (Citation)
Monthly	Nurse/Clinician	Adverse events	Common Terminology Criteria for Adverse Events
T0	Nurse	Clinical-anamnestic-demographic data	Registry card
Daily	Patients	Vital signs and main symptoms from patient-reported outcomes	Patient-reported outcomes
T0, T1, T2, T3	Patients	Patient-reported outcomes	Patient-reported outcomes
T0, T1, T2, T3	Nurse/Clinician	Performance status	Karnofsky performance status
T0, T1, T2, T3	Patients	Quality of life	Quality of Life SF-36
T1, T2, T3	Patients	System satisfaction and usability	SUS—System Usability Scale
T0, T1, T2, T3	Patients	Supportive care needs	Supportive care needs
T0, T1, T2, T3	Patients	Therapeutic adherence	MARS-5I

T0: baseline; T1: 30 days; T2: 90 days; T3: 180 days; SF: short form; MARS-5I: Medication Adherence Report Scale Italian.

**Table 2:** Participants’ timeline.

Timeline (People Involved)	T0 Baseline	T1 Month 1	Month 2	T2 Month 3	Month 4	Month 5	T3 Month 6
			**INTERVENTION ARM**				
enrolment (nurses and oncologists)	√						
randomisation (staff MSCT)	√						
follow-up visits (nurses and oncologists)		√	√	√	√	√	√
telenursing interventions (nurses)	√	√	√	√	√	√	√
monthly measurement (patients, nurses and oncologists)	√	√	√	√	√	√	√
			**CONTROL ARM**				
enrollment (nurses and oncologists)	√						
randomisation (staff MSCT)	√						
follow-up visits (oncologists)		√	√	√	√	√	√
monthly measurement (patients and oncologists)	√	√	√	√	√	√	√

MSCT: Monitoring and Supporting Clinical Trials.

## Data Availability

The datasets generated and analysed during the current study will be available from the corresponding author upon reasonable request.

## Supplementary Materials

The following supporting information can be downloaded at https://www.mdpi.com/article/10.3390/mps7050078/s1, Supplementary Table S1. Standard Protocol Items Recommendations for Interventional Trials (SPIRIT) checklist.

## Public Involvement Statement

Patients were involved in the data collection phase of the study.

## Guidelines and Standards Statement

The Standard Protocol Items Recommendations for Interventional Trials (SPIRIT) guidelines were used for the reporting (Chan, A.; Tetzlaff, J.M.; Gøtzsche, P.C.; Altman, D.G.; Mann, H.; Berlin, J.A.; Dickersin, K.; Hróbjartsson, A.; Schulz, K.F.; Parulekar, W.R.; Krleza-Jeric, K.; Laupacis, A.; Moher, D. SPIRIT 2013 explanation and elaboration: guidance for protocols of clinical trials. BMJ 2013, 346, e7586, 10.1136/bmj.e7586).
